# Retraction: Beyond the Curriculum: Exploring Perception of Equity, Diversity, and Inclusion Among Medical Students

**DOI:** 10.7759/cureus.r208

**Published:** 2025-12-05

**Authors:** Rehab A Alsaleh, Noha M Abd El-Fadeal, Nada M Alamoudi, Shereen El Tarhouny

**Affiliations:** 1 Department of Obstetrics and Gynaecology, Ibn Sina National College for Medical Studies, Jeddah, SAU; 2 Department of Biochemistry, Ibn Sina National College for Medical Studies, Jeddah, SAU; 3 Department of Medical Biochemistry and Molecular Biology, Faculty of Medicine, Suez Canal University, Ismailia, EGY; 4 Department of Medicine and Surgery, Ibn Sina National College for Medical Studies, Jeddah, SAU; 5 Health Professions Education Center, Ibn Sina National College for Medical Studies, Jeddah, SAU; 6 Department of Medical Biochemistry, Zagazig University, Zagazig, EGY

The Editors-in-Chief have retracted this article due to significant concerns about the accuracy of the statistical results in Tables 2, 3, and 4. Several values were found to be internally inconsistent or mathematically incompatible, including implausible standard deviations and incorrect relationships between t- and p-values that undermine confidence in the analyses. The authors disagree with the decision to retract.

